# Age-Related Increases in Benign Paroxysmal Positional Vertigo Are Reversed in Women Taking Estrogen Replacement Therapy: A Population-Based Study in Taiwan

**DOI:** 10.3389/fnagi.2017.00404

**Published:** 2017-12-12

**Authors:** Ding-Hao Liu, Chia-Hua Kuo, Chia-To Wang, Ch-Chih Chiu, Tzeng-Ji Chen, De-Kuang Hwang, Chung-Lan Kao

**Affiliations:** ^1^Department of Physical Medicine and Rehabilitation, Taipei Veterans General Hospital, Taipei, Taiwan; ^2^Institute of Clinical Medicine, National Yang-Ming University, Taipei, Taiwan; ^3^Department of Sports Sciences, University of Taipei, Taipei, Taiwan; ^4^Department of Physical Medicine and Rehabilitation, Taipei Veterans General Hospital, Yilan, Taiwan; ^5^Department of Family Medicine, Taipei Veterans General Hospital, Taipei, Taiwan; ^6^Institute of Hospital and Health Care Administration, National Yang-Ming University, Taipei, Taiwan; ^7^Department of Ophthalmology, Taipei Veterans General Hospital, Taipei, Taiwan; ^8^Department of Ophthalmology, School of Medicine, National Yang-Ming University, Taipei, Taiwan; ^9^Department of Physical Medicine and Rehabilitation, School of Medicine, National Yang-Ming University, Taipei, Taiwan

**Keywords:** benign paroxysmal positional vertigo, estrogen, hormone replacement, menopause, population based study

## Abstract

Benign paroxysmal positional vertigo (BPPV) is the most common cause of peripheral vertigo. Numerous investigations have reported an increased BPPV incidence in females and in the aged population. The hormonal characteristics of BPPV patients have not been previously investigated. This study aimed to determine the risk of BPPV in relation to menopause in a population-based study.

**Materials and Methods**: This retrospective population-based study was designed to use a nationwide longitudinal health insurance database to follow and analyze the incidence of and protective factors against BPPV in a Taiwanese population.

**Data Analyses**: Univariate and multivariate analyses were performed to calculate the adjusted hazard ratio (aHR) for the incidence of BPPV using Cox-proportional regression models.

**Results**: In the multivariate analyses, we found that older people (older than 65 years old) were more prone to develop BPPV (aHR: 5.37, 95% CI: 0 4.83–5.97, *p* < 0.001). The risk of BPPV was analyzed in two specific age subgroups of elderly females. Results revealed that in both age groups (45–65 years old and >65 years old), patients who took estrogen for menopausal syndromes had a significantly lower incidence of BPPV (aHR; 0.01, 95% CI: 0.06–0.23, *p* < 0.001).

**Conclusion**: Our study provides a novel etiology and possible treatment method for the prevention of BPPV. Further studies may focus on the pathophysiological mechanism of estrogen in BPPV patients and the development of new drugs for the prevention and treatment of BPPV.

## Introduction

Benign paroxysmal positional vertigo (BPPV) is the most common dizziness problems. BPPV is characterized by brief period of vertiginous attacks precipitated by changes in head position (Bárány, 1920) and causes balance disorders in elderly patients (Baloh, 1992). Utricular otoconia have been reported to separate from the mass and dislocate to the semicircular canals. Then, the canals become sensitized and generate inaccurate information, causing BPPV (Schuknecht, 1969; Hall et al., 1979). We made diagnosis of BBPV by clinical observations and nystagmus elicited by Dix–Hallpike test (Dix and Hallpike, 1952; Furman and Cass, 1999).

The etiology of BPPV remains unclear. In our previous investigation, BPPV was an independent risk factor of subsequent ischemic stroke (Kao et al., 2014). Furthermore, aging, brain trauma, sedentary life style, viral labyrinthitis, and the anterior vestibular artery occlusion may contribute population to BPPV (Baloh et al., 1987; Steenerson et al., 2005). BPPV can cause physical and psychological distress due to attacks. Comorbidities, such as, depression, anxiety, and chronic dizziness, may be undervalued, particularly in females (Ferrari et al., 2014).

Menopause is defined as the absence of menstrual cycles and ovarian activity for more than 12 months. Women may experience sleep disorders, night sweats, mood fluctuation, and vasomotor instability. Long-term estrogen deficiency may result in severe complications, including cardiovascular diseases and osteoporosis. Abundant investigations have presented an increased BPPV incidence in women and the elderly individuals (Lynn et al., 1995; Angeli et al., 2003; Yimtae et al., 2003; Steenerson et al., 2005; Kao et al., 2009). Ogun et al. (2014a) suggested that hormonal fluctuations (particularly during menopause) may incline to develop BPPV. Park and Viirre (2010) proposed that unstable levels of ovarian neurosteroids during the perimenopausal period might trigger vestibular migraine.

The hormonal characteristics of BPPV patients have not been comprehensive explored. Therefore, this study aimed to investigate the risk of BPPV in relation to menopause in a population-based study. This retrospective study may provide useful information concerning to the demographic risk factors of BPPV and its connection with hormone replacement therapy in Taiwanese populations.

## Materials and methods

### Study design

This retrospective population-based study was designed to use a nationwide longitudinal health insurance database to follow and analyze the incidence of and protective factors against BPPV in a Taiwanese population. The study was approved by the Institutional Review Board of National Yang-Ming University, Taiwan (YM105080E).

The National Health Insurance (NHI) program in Taiwan was established in 1995. It is a single-payer, mandatory medical care system covering more than 99% of residents and medical utilities in Taiwan. One million registered participants were randomly selected from the database of this program in 2000. These subjects represented ~4% of all citizens in Taiwan at that time. All claims data between 1997 and 2011 were collected from all sampled subjects.

### Inclusion and exclusion criteria

When calculating the incidence of BPPV, all subjects in the database were included, and all claims data were reviewed. Subjects with abnormal registry claim data, such as unknown gender or unknown birthdate, were excluded. Subjects younger than 15 years old were excluded. Subjects with any previous diagnosis of endometrial or breast cancer were excluded from the analysis.

### Incidence of BPPV

Subjects were defined as having BPPV when more than 2 consensus International Classification of Disease, Ninth Revision, Clinical Modification (ICD-9-CM) diagnostic codes of BPPV (386.11) were identified from the database. The first date when the patient was diagnosed with BPPV in the database was defined as their incident time. The annual incidence was calculated as the number subjects with newly diagnosed BPPV in a particular year divided by the total population not diagnosed before the first day of that year. The annual prevalence was calculated as the number of subjects with a diagnosis of BPPV divided by the total registered population in that year. The age group-stratified incidence and prevalence were also calculated.

To avoid the misclassification of prevalent cases as incident cases, data obtained before January 1, 2000 were excluded from the table and subsequent analysis.

### Risk factors for BPPV

Patients diagnosed prior to January 1, 2000, were excluded from the analysis of the risk factors for BPPV. All subjects were followed up from January 1, 2000, to the date when the first diagnostic code relevant to BPPV was identified or to the last date when the last participant in the database was identified. Age was then stratified in to four groups: 15–25 years old, 25–45 years old, 45–65 years old, and older than 65 years.

Urbanization of insurance regions and insurance wages were used as indicators of socioeconomic status. Urbanization of insurance regions is an important indicator of socioeconomic status in Taiwan. This factor was classified into three categories (urban, suburban, and rural) based on population density, medical resources, age, and education levels in the areas. Seventy regions were defined as urban, 144 regions were defined as suburban, and 96 regions were defined as rural areas in Taiwan based on these criteria. In Taiwan, the insurance wages of individuals are defined based on their occupations and monthly incomes. The subjects' insurance wages were categorized as equal to or greater than NT $40,000 (US $1,250), between NT $20,000 and NT $40,000 (US $625–1,250), lower than NT $20,000 (US $625), and fixed-premium if the participants' insurance types were dependent, farmer, fisher, or other fixed-premium types. However, the degree of health care utilization was categorized into four groups based on the total numbers of ambulatory and in-patient claims that were not related to BPPV. The comorbidities of BPPV such like anxiety disorder (Chen et al., 2016), hypertension, type 2 diabetes mellitus, and hyperlipidemia were all included as covariate (De Stefano et al., 2014).

A subgroup of elderly females was analyzed after being divided into two groups (age between 45 and 65 years, as the para-menopausal stage, and older than 65 years, as the post-menopausal stage). The database was searched to determine whether the subject had been prescribed estrogen before the BPPV diagnosis, and these data were analyzed during the subgroup analyses. The type of estrogen therapy in our study is conjugated equine estrogens (CEE) and oral estradiol (E2).

### Data analyses

Univariate and multivariate analyses were performed to calculate the adjusted hazard ratio (aHR) for the incidence of BPPV using Cox-proportional regression models. The SAS statistical package version 9.2 (SAS Inc., Cary, North Carolina, USA) was used for all estimations. The 95% confidence intervals (CIs) of the aHRs were calculated, and the two-sided significance level was set at 0.05.

## Results

A total of 7,200 BPPV patients were included in the analysis. The average age of the BPPV patients in the study was 51.81 ± 16.1 years old. Of the BPPV patients, 63.30% were female. A total of 55.3% of the BPPV patients lived in urban regions, according to the geographic distribution of Taiwan. The fixed-premium type of insurance wage accounted for the largest percentage (46.7%) of BPPV patients. The comorbidities such as hypertension (48.2%), cardiovascular disease (26.2%), diabetes mellitus (19%), dyslipidemia (30.5%), and anxiety disorder (36.8%) were presented, respectively. The demographic data of the BPPV patients are shown in Table 1.

**Table 1:** Baseline characteristics of BPPV patients.

**BPPV Patients *N* = 7,200**			
Age	Mean ± *SD*	51.81	16.1^***^
Gender (M)	*n*, %	2,640	36.70%^***^
Gender (F)	*n*, %	4,560	63.30%
Urban (Urban)	*n*, %	3,980	55.30%^***^
Urban (Suburban)	*n*, %	2,400	33.30%
Urban (Rural)	*n*, %	820	11.40%
Insurance Wage (≥NT$40,000)	*n*, %	491	6.80%
Insurance Wage (NT$20,000–40,000)	*n*, %	814	11.30%
Insurance Wage (<NT$20,000)	*n*, %	2,533	35.20%
Insurance Wage (Fixed)	*n*, %	3,362	46.70%
Hypertension	*n*, %	3,467	48.20%^***^
Cardiovascular disease	*n*, %	1,889	26.20%^***^
Diabetes mellitus	*n*, %	1,365	19.00%^***^
Dyslipidemia	*n*, %	2,198	30.50%^***^
Anxiety disorder	*n*, %	2,649	36.80%^***^

*** *p < 0.001 indicates statistically significant*.

**Figure 1:**
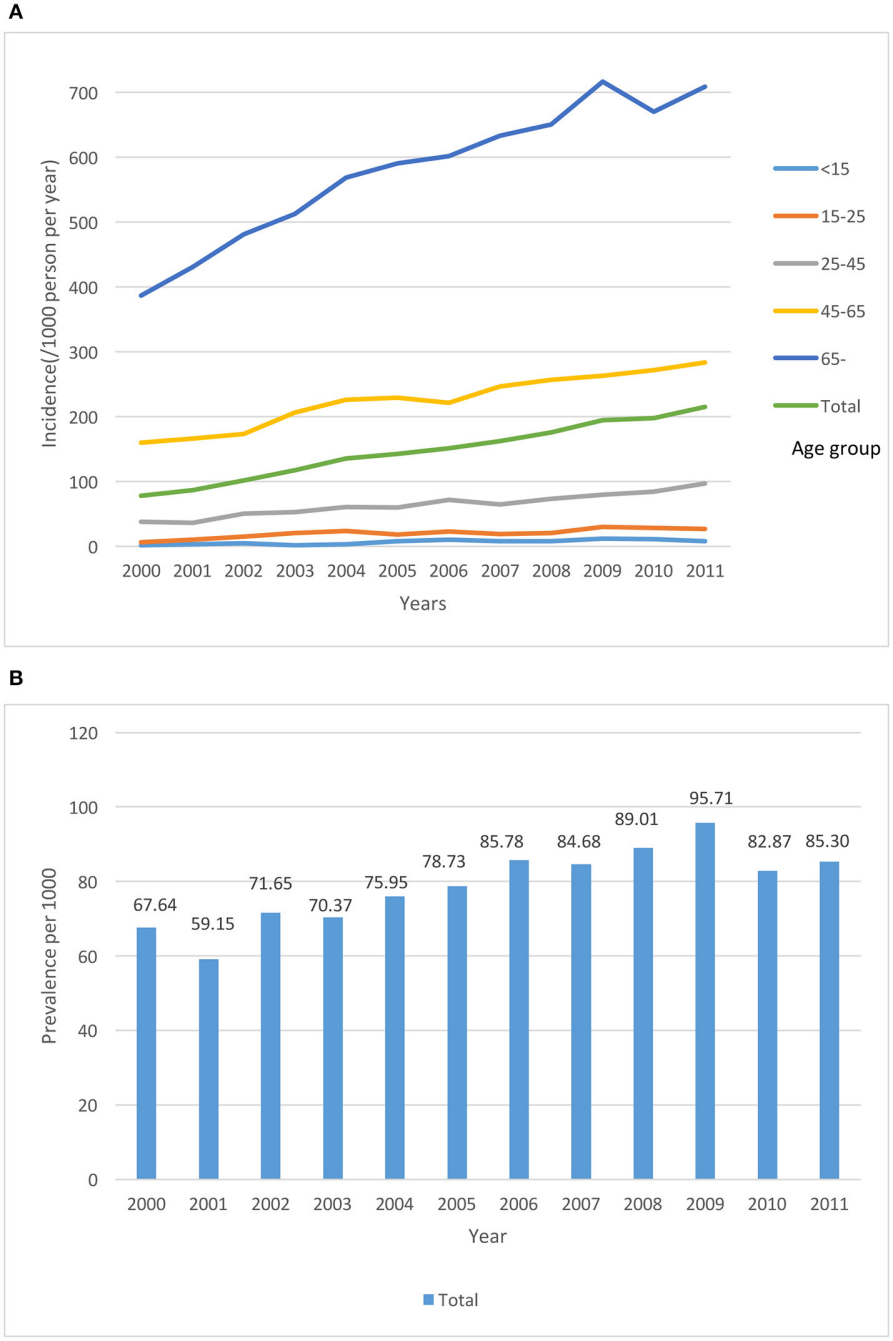
The incidence **(A)** and prevalence **(B)** of BPPV in the different age groups from 2000 to 2011.

All 7,200 patients were followed until 2011. The incidence and prevalence of BPPV in the different age groups from 2000 to 2011 is shown in Figure 1. To further evaluate the risk of developing BPPV among population with different characteristics, a subgroup analysis stratified by gender was performed. Table 2 shows the risk factors for BPPV according to gender groups. In the multivariate analyses, we found that older people (older than 65 years old) were more prone to develop BPPV (aHR: 5.37, 95% CI; 4.83–5.97, *p* < 0.001). Interestingly, females living in rural areas were found have an increased risk of BPPV (aHR; 1.17, 95% CI; 1.06–1.29, *p* < 0.001). Individuals with greater health care utilization had a higher incidence of BPPV diagnoses (aHR; 11.32, 95% CI; 9.76–13.13, *p* < 0.001). Furthermore, male and female gender groups showed similar health care utilization rates. Only males with an insurance wage between NT $20,000 and 40,000 had a high risk of BPPV (aHR; 1.18, 95% CI; 1.02–1.137, *p* < 0.05); the other insurance wage categories did not have a significant risk.

**Table 2:** Multivariable regression analysis of the incidence of BPPV.

	**Total**			**Male**	**Female**
**Characteristics**	**HR**	***p*-value^a^**	**aHR (95% CI)**	**aHR (95% CI)**	**aHR (95% CI)**
Gender (Female/Male)	1.8	<0.001	1.43 (1.36–1.5)^***^		
**AGE**					
15–25	1 (ref)		1 (ref)	1 (ref)	1 (ref)
25–45	2.20	<0.001	1.91 (1.72–2.13)^***^	1.87 (1.5–2.27)^***^	1.94 (1.7–2.2)^***^
45–65	6.48	<0.001	3.91 (3.52–4.34)^***^	3.96 (3.27–4.8)^***^	3.90 (3.44–4.42)^***^
>65	11.40	<0.001	5.37 (4.83–5.97)^***^	7.15 (5.92–8.63)^***^	4.50 (3.95–5.13)^***^
**URBAN**					
Urban	1 (ref)		1 (ref)	1 (ref)	1 (ref)
Suburban	1.19	<0.001	1.14 (1.09–1.2)^***^	1.14 (1.05–1.24)^**^	1.15 (1.08–1.23)^***^
Rural	1.42	<0.001	1.11 (1.02–1.2)^*^	1.01 (0.88–1.16)	1.17 (1.06–1.29)^***^
**HEALTH CARE UTILIZATION**					
Low	1 (ref)		1 (ref)	1 (ref)	1 (ref)
Mid–Low	2.89	<0.001	2.70 (2.3–3.18)^***^	2.75 (2.08–3.65)^***^	2.57 (2.17–3.05)^***^
Mid–High	6.94	<0.001	5.38 (4.62–6.27)^***^	5.68 (4.37–7.37)^***^	4.56 (3.88–5.35)^***^
High	20.23	<0.001	11.32 (9.76–13.13)^***^	12.22 (9.46–15.78)^***^	8.65 (7.4–10.11)^***^
**INSURANCE WAGE**					
≥40,000	1 (ref)		1 (ref)		
2000–40,000	1.08	0.2	1.08 (0.97–1.21)	1.18 (1.02–1.37)^*^	0.93 (0.78–1.11)
<20,000	1.12	0.03	1.06 (0.96–1.17)	0.96 (0.95–1.24)	0.95 (0.82–1.11)
Fixed	1.08	0.1	1.1 (0.99–1.22)	0.99 (0.98–1.28)	1.00 (0.85–1.17)

aHR, adjusted hazard ratio;

*ref, reference*.

a *Using Cox-proportional regression models*.

* p < 0.05;

** p < 0.01;

*** *p < 0.001 indicates statistically significant*.

The risk of BPPV was analyzed in two specific age subgroups of elderly females (i.e., “para-menopausal stage” and “post-menopausal stage”) (Table 3). We found that in both age groups (45–65 years old and >65 years old), patients who took estrogen for menopausal syndromes had a significantly lower incidence of BPPV (aHR; 0.01, *p* < 0.001).

**Table 3:** Incidence of BPPV (Females).

	**Age 45–65 yr**.		**Age** >**65 yr**.	
**Characteristics**	**aHR**	***p*-value^a^**	**aHR**	***p*-value^a^**
Age	1.03	0.14	0.9	0.09
**URBAN**				
Urban	1 (ref)		1 (ref)	
Suburban	1.30	0.26	3.10	0.11
Rural	1.54	0.22	4.07	0.06
**INSURANCE WAGE**				
≥NT$40,000	1(ref)		1(ref)	
NT$20,000–40,000	0.24^*^	0.02	1.32	0.99
<NT$20,000	0.49	0.08	10,391	0.96
Fixed	0.58	0.21	15,382	0.96
**HEALTH CARE UTILIZATION**				
Low	1 (ref)		1 (ref)	
Mid-Low	2.48	0.24	2,413	0.94
Mid-High	4.48^*^	0.04	5,540	0.93
High	6.52^*^	0.01	9,215	0.3
Estrogen prescription (Yes/No)	0.01^***^	<0.001	0.01^***^	<0.001

aHR, adjusted hazard ratio;

*ref, reference*.

a *Using Cox-proportional regression models*.

* p < 0.05;

*** *p < 0.001 indicates statistically significant*.

## Discussion

Our study results provided clinical information about BPPV. The mean age of BPPV patients in the study was 52 years old, and females exhibited a higher incidence of BPPV than males. These characteristic findings were similar to those of a previous cohort study (von Brevern et al., 2007). Our 1-year prevalence and 1-year incidence of BPPV were 0.16 and 0.06%, respectively (Figure 1). Compared to a previous study by Von Brevern et al. that found a BPPV incidence rate of 0.6%/year, our result (~0.06%) is only slightly higher than one-tenth of the value reported. The difference between our data and the previous study may be due to the following reasons. First, our data reflect the treated incidence of BPPV. Only individuals with more severe dizziness visit clinics. Additionally, only physicians in specialties related to dizziness, such as neurologists or otolaryngologists, diagnosed BPPV. Second, the main purpose of codes in the NHI database is for insurance payments. Some comorbidities will have crowding-out effect among the codes due to the application of premium subsidies. Finally, the differences in BPPV between Caucasians and Asians have never been documented; therefore, further large-scale demographic studies must be conducted to determine these differences.

Interestingly, the 1-year incidence was exceedingly high in 2008 and 2009 (0.089 and 0.095%, respectively). On possible reason for this increase could be the 2008 world financial crisis. The financial crisis affected the health status of many individuals (Zavras et al., 2016). Increased diagnoses of tinnitus and vertigo were reported in Greece after the financial crisis (Karatzanis et al., 2012). Furthermore, a recent systematic review determined that the proportion of suicides increased and mental health worsened during the crisis (Parmar et al., 2016). An increased proportion of psychiatric disorders has been reported among patients with vertigo syndromes and has been attributed to vestibular dysfunction (Eckhardt-Henn et al., 2008). In addition, patients with anxiety disorders tend to develop BPPV (Chen et al., 2016). Therefore, the increased mental distress in 2008 may be related to the high incidence rate of BPPV.

The risk factors for BPPV were examined in stratified group analyses. Old age is one significant risk factor among all groups. Several studies have demonstrated an increased incidence of BPPV among aging population (Neuhauser, 2007; von Brevern et al., 2007). Vestibular function decline has been shown to associate with the age-related degenerate in the number of neurons and vestibular hair cells (Iwasaki and Yamasoba, 2015). Urbanization of insurance regions and insurance wages were used as the indicators of socioeconomic status. Female patients living in rural areas was a risk factor for BPPV according to the multivariate regression. Lower socioeconomic status was associated with the development of BPPV. Consistent with previous epidemiologic studies, Neuhauser and Agrawal demonstrated that a lower education level and socioeconomic status were associated with vestibular vertigo (Neuhauser et al., 2005; Agrawal et al., 2009).

The high health care utilization patient group had a ~20 times higher risk of having BPPV than the group with low health care utilization. The likely reason is that the symptoms of BPPV are extremely uncomfortable. Patients who suffer from rotational vertigo, nausea, and vomiting may seek medical support immediately. Recurrent BPPV can also contribute a higher rate of medical utilization.

**Figure 2:**
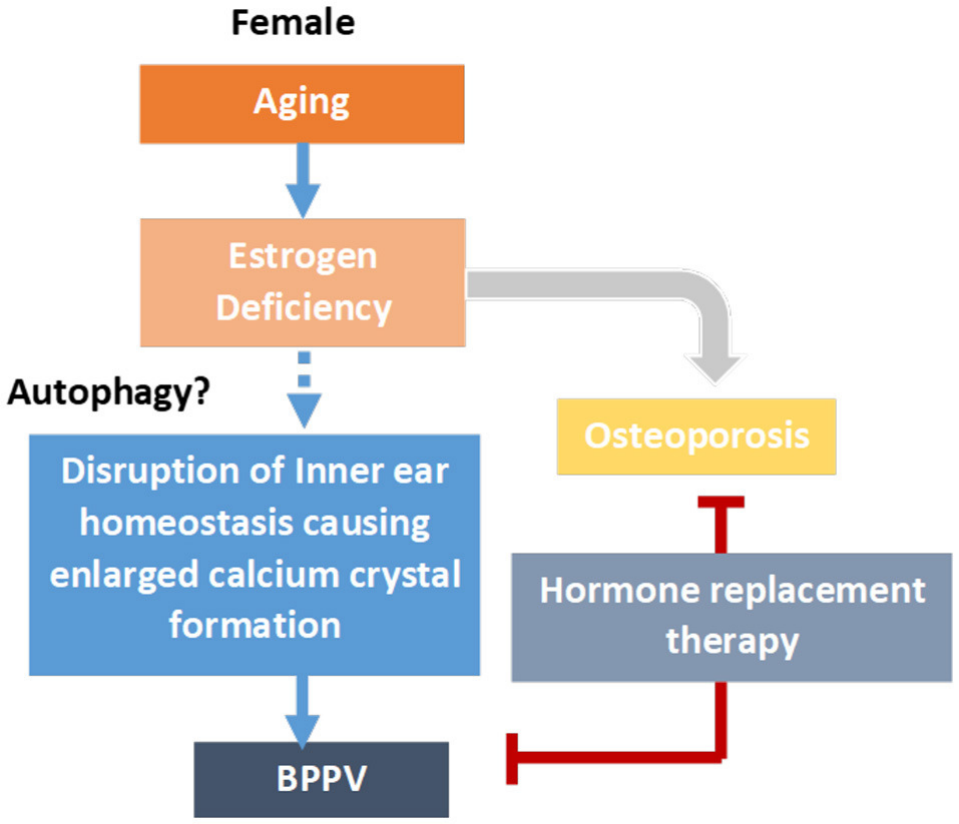
Proposed mechanism for estrogen replacement and the incidence of BPPV. Aging causes estrogen deficiency in para-menopausal and post-menopausal women. Estrogen replacement may reduce to progression of osteoporosis, and at the same time, possibly reduce BPP formation. The disruption of inner ear otoconia biogenesis homeostasis, possibly caused by reduced autophagic activities, may cause enlargement of calcium crystals in the inner ears, leading to BPPV formation.

As previously mentioned, an older age and female preponderance were found among BPPV patients. Some authors have suggested that estrogen may play a role in the formation of BPPV (Yamanaka et al., 2013; Ogun et al., 2014a). To date, the relationship between estrogen and BPPV remains unclear. A previous animal study found that ovariectomy affects otoconia morphology in rats (Lynn et al., 1995). Recently, in a population-based study, Ogun, et al. found that hormonal fluctuations (particularly during menopause) may increase the trend to develop BPPV (Ogun et al., 2014a). The likely pathophysiology is related to the estrogen receptors in the inner ear, primarily the vestibular dark cells in the ampulla and utricle; estrogen may also affect the ionic and anionic homeostasis of endolymph by regulating the expression of ion and anion channels and pumps (Lee and Marcus, 2001; Chen and Nathans, 2007). Ogun et al. speculated that a sudden decrease or increase in estrogen would disrupt electrical charge homeostasis and influence neurosensory function (Ogun et al., 2014b). Marino et al. (2010) found that reduced autophagic activity may cause unbalanced homeostasis of otoconial biogenesis in Atg4b null mice, causing enlarged calcium crystals formation. Further investigation on the relationship between the autophagic activities in inner ears and estrogen deficiency requires further investigation. The proposed mechanism for the relationship between BPPV and menopause is shown in Figure 2.

One novel finding of our study was that females who took estrogen for menopause syndrome had a significantly lower incidence of BPPV. A previous study reported that impaired postural balance was correlated with estrogen insufficient after menopause (Winner et al., 1989; Hammar et al., 1996; Naessen et al., 1997). Moreover, the perception of disturbances in postural balance has been linked to the frequency of vasomotor symptoms (Ekblad et al., 2000). Accordingly, Naessen et al. found that starting hormone therapy soon after menopause ameliorated postural balance to levels normally observed in young female subjects (Naessen et al., 2007). Similar findings were reported by Coksuer et al., the vertigo symptom gone after took hormone therapy (Coksuer et al., 2011). Furthermore, Yang et al. conducted an animal study and the result showed the bilateral ovariectomy rat accepted female sex hormone replacement therapy were reversed the decrease of otoconin 90 levels (Yang et al., 2017). Otoconin 90 acts as main protein to maintain the normal morphology and growth of otoconia (Petko et al., 2008). The epigenetic regulation of estrogen on Otoconin 90 may need further investigation.

These findings might support our results that estrogen replacement therapy can reduce the incidence of BPPV in menopausal women. We can speculate that a sufficient and more stable estrogen blood level may provide protective effects, according to our study results. However, the effects of estrogen on autophagic activity and the possible influence of epigenetic regulation require further investigation.

Another explanation for a higher BPPV incidence in the older female population is its relationship with osteoporosis. A systematic review study indicated that BPPV is associated with osteoporosis (Yu et al., 2014). One recent study reported that vitamin D deficiency and decreased ionized Ca levels may be risk factors for BPPV (Kim et al., 2016). However, the concurrence of two morbidities is not encouraged of a relationship (Talaat et al., 2015). Our findings provide an explanation other than Ca homeostasis for this issue. Estrogen deficiency is the major predisposing factor to bone loss after menopause. Selective estrogen receptor modulators (SERMs) have been widely used to treat post-menopausal osteoporosis. According to our study, loss of estrogen protection is a risk factor of BPPV. Therefore, further research on the relationship between estrogen and BPPV may help prevent the recurrence and occurrence of BPPV.

One strength of this study is its nationwide population setting; its wide age range and numerous sample size provide satisfactory power for hazard ratio analyses. Moreover, our risk factor analysis had a cohort design and was performed through the assessment of a reliable insurance system. Nevertheless, our study has some limitations. First, the diagnosis of BPPV in this study primarily depended on the ICD codes recorded in the NHI database. Even though we have included only subjects who were diagnosed with BPPV at least twice in outpatient clinics to reduce the bias of over/under diagnosis, the exact number of BPPV patients is still different from a prospective study design. Second, our database primarily consists of Taiwanese population, therefore, our results may not explain the racial difference in relationship between BPPV and estrogen. Last, due to the lack of clinical data, we couldn't obtain the exact hormone level or status of menopause syndrome of subjects.

Our study provides a novel etiology and possible treatment method for the prevention of BPPV. Further studies may focus on the pathophysiological mechanism of estrogen in BPPV patients and the development of new drugs for the prevention and treatment of BPPV.

## Author contributions

D-HL: conceiving, drafting and graphing, data analysis; C-HK: drafting, data analysis, revised; C-TW: data collection, analysis; C-CC: drafting and graphing; T-JC: data collection, mining; D-KH: data mining, data analysis, statistical analysis; C-LK: conceiving designing, drafting, data analysis, critically revised of this study. All authors read and approved the final manuscript.

### Conflict of interest statement

The authors declare that the research was conducted in the absence of any commercial or financial relationships that could be construed as a potential conflict of interest.

## References

[B1] Agrawal Y. Carey J. P. Della Santina C. C. Schubert M. C. Minor L. B. (2009). Disorders of balance and vestibular function in US adults: data from the National Health and Nutrition Examination Survey, 2001-2004. Arch. Intern. Med. 169, 938–944. 10.1001/archinternmed.2009.66 19468085

[B2] Angeli S. I. Hawley R. Gomez O. (2003). Systematic approach to benign paroxysmal positional vertigo in the elderly. Otolaryngol. Head Neck Surg. 128, 719–725. 10.1016/S0194-5998(03)00256-0 12748567

[B3] Baloh R. W. (1992). Dizziness in older people. J. Am. Geriatr. Soc. 40, 713–721. 10.1111/j.1532-5415.1992.tb01966.x 1607589

[B4] Baloh R. W. Honrubia V. Jacobson K. (1987). Benign positional vertigo: clinical and oculographic features in 240 cases. Neurology 37, 371–378. 10.1212/WNL.37.3.371 3822129

[B5] Bárány E. (1920). Diagnose yon krankheitserscheinungen im bereiche des otolithenapparates. Acta Otolaryngol. 2, 434–437. 10.3109/00016482009123103

[B6] Chen J. Nathans J. (2007). Estrogen-related receptor beta/NR3B2 controls epithelial cell fate and endolymph production by the stria vascularis. Dev. Cell. 13, 325–337. 10.1016/j.devcel.2007.07.011 17765677

[B7] Chen Z. J. Chang C. H. Hu L. Y. Tu M. S. Lu T. Chen P. M.. (2016). Increased risk of benign paroxysmal positional vertigo in patients with anxiety disorders: a nationwide population-based retrospective cohort study. BMC Psychiatry 16:238. 10.1186/s12888-016-0950-2 27416989 PMC4946194

[B8] Coksuer H. Koplay M. Oghan F. Coksuer C. Keskin N. Ozveren O. (2011). Effects of estradiol–drospirenone hormone treatment on carotid artery intima-media thickness and vertigo/dizziness in postmenopausal women. Arch. Gynecol. Obstet. 283, 1045–1051. 10.1007/s00404-010-1487-0 20443014

[B9] De Stefano A. Dispenza F. Suarez H. Perez-Fernandez N. Manrique-Huarte R. Ban J. H.. (2014). A multicenter observational study on the role of comorbidities in the recurrent episodes of benign paroxysmal positional vertigo. Auris Nasus Larynx 41, 31–36. 10.1016/j.anl.2013.07.007 23932347

[B10] Dix M. R. Hallpike C. S. (1952). The pathology symptomatology and diagnosis of certain common disorders of the vestibular system. Proc. R. Soc. Med.U.S.A. 45, 341–354. 10.1177/000348945206100403 14941845 PMC1987487

[B11] Eckhardt-Henn A. Best C. Bense S. Breuer P. Diener G. Tschan R.. (2008). Psychiatric comorbidity in different organic vertigo syndromes. J. Neurol. 255, 420–428. 10.1007/s00415-008-0697-x 18338198

[B12] Ekblad S. Bergendahl A. Enler P. Ledin T. Mollen C. Hammar M. (2000). Disturbances in postural balance are common in postmenopausal women with vasomotor symptoms. Climacteric 3, 192–198. 10.1080/13697130008500097 11910621

[B13] Ferrari S. Monzani D. Baraldi S. Simoni E. Prati G. Forghieri M. (2014). Vertigo “in the pink”: the impact of female gender on psychiatric-psychosomatic comorbidity in benign paroxysmal positional vertigo patients. Psychosomatics 55, 280–288. 10.1016/j.psym.2013.02.005 23756120

[B14] Furman J. M. Cass S. P. (1999). Benign paroxysmal positional vertigo. N. Engl. J. Med. 341, 1590–1596. 10.1056/NEJM199911183412107 10564690

[B15] Hall S. F. Ruby R. R. McClure J. A. (1979). The mechanics of benign paroxysmal vertigo. J. Otolaryngol. 8, 151–158. 430582

[B16] Hammar M. L. Lindgren R. Berg G. E. Moller C. G. Niklasson M. K. (1996). Effects of hormonal replacement therapy on the postural balance among postmenopausal women. Obstet. Gynecol. 88, 955–960. 10.1016/S0029-7844(96)00356-0 8942834

[B17] Iwasaki S. Yamasoba T. (2015). Dizziness and imbalance in the elderly: age-related decline in the vestibular system. Aging Dis. 6, 38–47. 10.14336/AD.2014.0128 25657851 PMC4306472

[B18] Kao C. L. Cheng Y. Y. Leu H. B. Chen T. J. Ma H. I. Chen J. W.. (2014). Increased risk of ischemic stroke in patients with benign paroxysmal positional vertigo: a 9-year follow-up nationwide population study in Taiwan. Front. Aging Neurosci. 6:108. 10.3389/fnagi.2014.00108 24917815 PMC4040439

[B19] Kao C. L. Hsieh W. L. Chern C. M. Chen L. K. Lin M. H. Chan R. C. (2009). Clinical features of benign paroxysmal positional vertigo (BPPV) in Taiwan: differences between young and senior age groups. Arch. Gerontol. Geriatr. 49(Suppl. 2), S50–S54. 10.1016/S0167-4943(09)70014-7 20005428

[B20] Karatzanis A. D. Symvoulakis E. K. Nikolaou V. Velegrakis G. A. (2012). Potential impact of the financial crisis on outpatient hospital visits due to otorhinolaryngologic disorders in Crete, Greece. Int. J. Med. Sci. 9, 126–128. 10.7150/ijms.3447 22253558 PMC3258553

[B21] Kim S. Y. Han S. H. Kim Y. H. Park M. H. (2016). Clinical features of recurrence and osteoporotic changes in benign paroxysmal positional vertigo. Auris Nasus Larynx 44, 156–161. 10.1016/j.anl.2016.06.006 27423924

[B22] Lee J. H. Marcus D. C. (2001). Estrogen acutely inhibits ion transport by isolated stria vascularis. Hear. Res. 158, 123–130. 10.1016/S0378-5955(01)00316-1 11506944

[B23] Lynn S. Pool A. Rose D. Brey R. Suman V. (1995). Randomized trial of the canalith repositioning procedure. Otolaryngol. Head Neck Surg. 113, 712–720. 10.1016/S0194-5998(95)70010-2 7501382

[B24] Marino G. Fernandez A. F. Cabrera S. Lundberg Y. W. Cabanillas R. Rodriguez F.. (2010). Autophagy is essential for mouse sense of balance. The Journal of clinical investigation. 120, 2331–2344. 10.1172/JCI42601 20577052 PMC2898610

[B25] Naessen T. Lindmark B. Lagerstrom C. Larsen H. C. Persson I. (2007). Early postmenopausal hormone therapy improves postural balance. Menopause 14, 14–19. 10.1097/01.gme.0000248707.53075.7f 17091024

[B26] Naessen T. Lindmark B. Larsen H. C. (1997). Better postural balance in elderly women receiving estrogens. Am. J. Obstet. Gynecol. 177, 412–416. 10.1016/S0002-9378(97)70207-2 9290460

[B27] Neuhauser H. K. (2007). Epidemiology of vertigo. Curr. Opin. Neurol. 20, 40–46. 10.1097/WCO.0b013e328013f432 17215687

[B28] Neuhauser H. K. von Brevern M. Radtke A. Lezius F. Feldmann M. Ziese T.. (2005). Epidemiology of vestibular vertigo: a neurotologic survey of the general population. Neurology 65, 898–904. 10.1212/01.wnl.0000175987.59991.3d 16186531

[B29] Ogun O. A. Buki B. Cohn E. S. Janky K. L. Lundberg Y. W. (2014a). Menopause and benign paroxysmal positional vertigo. Menopause 21, 886–889. 10.1097/GME.0000000000000190 24496089 PMC4110114

[B30] Ogun O. A. Janky K. L. Cohn E. S. Buki B. Lundberg Y. W. (2014b). Gender-based comorbidity in benign paroxysmal positional vertigo. PLoS ONE 9:e105546. 10.1371/journal.pone.0105546 25187992 PMC4154861

[B31] Park J. H. Viirre E. (2010). Vestibular migraine may be an important cause of dizziness/vertigo in perimenopausal period. Med. Hypotheses 75, 409–414. 10.1016/j.mehy.2009.04.054 20692105

[B32] Parmar D. Stavropoulou C. Ioannidis J. P. (2016). Health outcomes during the 2008 financial crisis in Europe: systematic literature review. BMJ 354:i4588. 10.1136/bmj.i4588 27601477 PMC5013230

[B33] Petko J. A. Millimaki B. B. Canfield V. A. Riley B. B. Levenson R. (2008). Otoc1: a novel otoconin-90 ortholog required for otolith mineralization in zebrafish. Dev. Neurobiol. 68, 209–222. 10.1002/dneu.20587 18000829 PMC2730775

[B34] Schuknecht H. F. (1969). Cupulolithiasis. Arch. Otolaryngol. 90, 765–778. 10.1001/archotol.1969.00770030767020 5353084

[B35] Steenerson R. L. Cronin G. W. Marbach P. M. (2005). Effectiveness of treatment techniques in 923 cases of benign paroxysmal positional vertigo. Laryngoscope 115, 226–231. 10.1097/01.mlg.0000154723.55044.b5 15689740

[B36] Talaat H. S. Abuhadied G. Talaat A. S. Abdelaal M. S. (2015). Low bone mineral density and vitamin D deficiency in patients with benign positional paroxysmal vertigo. Eur. Arch. Otorhinolaryngol. 272, 2249–2253. 10.1007/s00405-014-3175-3 24973969

[B37] von Brevern M. Radtke A. Lezius F. Feldmann M. Ziese T. Lempert T.. (2007). Epidemiology of benign paroxysmal positional vertigo: a population based study. J. Neurol. Neurosurg. Psychiatr. 78, 710–715. 10.1136/jnnp.2006.100420 17135456 PMC2117684

[B38] Winner S. J. Morgan C. A. Evans J. G. (1989). Perimenopausal risk of falling and incidence of distal forearm fracture. BMJ 298, 1486–1488. 10.1136/bmj.298.6686.1486 2503081 PMC1836686

[B39] Yamanaka T. Shirota S. Sawai Y. Murai T. Fujita N. Hosoi H. (2013). Osteoporosis as a risk factor for the recurrence of benign paroxysmal positional vertigo. Laryngoscope 123, 2813–2816. 10.1002/lary.24099 23568754

[B40] Yang H. Gu H. Sun W. Li Y. Wu H. Burnee M.. (2017). Estradiol deficiency is a risk factor for idiopathic benign paroxysmal positional vertigo in postmenopausal female patients. Laryngoscope. [Epub ahead of print]. 10.1002/lary.26628 28480516

[B41] Yimtae K. Srirompotong S. Srirompotong S. Sae-Seaw P. (2003). A randomized trial of the canalith repositioning procedure. Laryngoscope 113, 828–832. 10.1097/00005537-200305000-00011 12792318

[B42] Yu S. Liu F. Cheng Z. Wang Q. (2014). Association between osteoporosis and benign paroxysmal positional vertigo: a systematic review. BMC Neurol. 14:110. 10.1186/1471-2377-14-110 24886504 PMC4039044

[B43] Zavras D. Zavras A. I. Kyriopoulos I. I. Kyriopoulos J. (2016). Economic crisis, austerity and unmet healthcare needs: the case of Greece. BMC Health Serv. Res. 16:309. 10.1186/s12913-016-1557-5 27460938 PMC4962475

